# Effects of Flight on Gene Expression and Aging in the Honey Bee Brain and Flight Muscle

**DOI:** 10.3390/insects4010009

**Published:** 2012-12-20

**Authors:** Joseph W. Margotta, Georgina E. Mancinelli, Azucena A. Benito, Andrew Ammons, Stephen P. Roberts, Michelle M. Elekonich

**Affiliations:** 1 School of Life Sciences, University of Nevada, Las Vegas, NV 89154, USA; E-Mails: margotta@unlv.nevada.edu (J.W.M.); benitoa@unlv.nevada.edu (A.A.B.); 2 Department of Biology, Central Michigan University, Mount Pleasant, MI 48858, USA; E-Mails: manci1ge@cmich.edu (G.E.M.); rober2sp@cmich.edu (S.P.R.); 3 Department of Biological Sciences, Goshen College, Goshen, IN 46526, USA; E-Mail: aammons@goshen.edu

**Keywords:** honey bee, genomics, aging, flight

## Abstract

Honey bees move through a series of in-hive tasks (e.g., “nursing”) to outside tasks (e.g., “foraging”) that are coincident with physiological changes and higher levels of metabolic activity. Social context can cause worker bees to speed up or slow down this process, and foragers may revert back to their earlier in-hive tasks accompanied by reversion to earlier physiological states. To investigate the effects of flight, behavioral state and age on gene expression, we used whole-genome microarrays and real-time PCR. Brain tissue and flight muscle exhibited different patterns of expression during behavioral transitions, with expression patterns in the brain reflecting both age and behavior, and expression patterns in flight muscle being primarily determined by age. Our data suggest that the transition from behaviors requiring little to no flight (nursing) to those requiring prolonged flight bouts (foraging), rather than the amount of previous flight *per se*, has a major effect on gene expression. Following behavioral reversion there was a partial reversion in gene expression but some aspects of forager expression patterns, such as those for genes involved in immune function, remained. Combined with our real-time PCR data, these data suggest an epigenetic control and energy balance role in honey bee functional senescence.

## 1. Introduction

Aerobic cellular respiration inevitably produces reactive oxygen species (ROS) that damage DNA, proteins, and lipids if antioxidant and repair systems are overwhelmed [1,2]. These accumulated effects of oxidative stress are the basis for the free radical theory of aging, which is widely researched, but often disputed [3], (but see [4,5,6]). Studies in social insects reveal ROS damage is important in aging, but only a single part of a more complex phenomenon [7]. Despite the controversy among theories of aging, evidence suggests that damage to biological macromolecules readily leads to premature aging, cell death, and senescence [8] unless defense systems can be up-regulated. However, individuals with increased antioxidant capacity are not necessarily better prepared to mitigate damage from ROS [9]. Consequently, an organism’s ability to mitigate the effects of ROS changes ontogenetically across its lifetime and is affected by diet and other environmental conditions [10]. While metabolically-intensive behaviors and other secondary sexual traits requiring greater ability to mitigate ROS may be selected for as honest signals of fitness [10,11,12], how these physical and behavioral traits contribute to senescence is not well-known. Understanding how senescence occurs and how it is influenced by behavioral development and behavioral intensity may reveal how behavior itself can damage a cell and consequently limit lifespan.

Few studies link variation in metabolically-intensive, naturally-occurring behaviors to oxidative stress, fitness, and lifespan [10]. In this study, we use the experimental tractability of the honey bee (*Apis mellifera*), to examine links between behavior, oxidative stress, and senescence. Senescence in traditional model organisms such as mice, rats, flies, and nematodes is characterized by irreversible aging as time progresses, while honey bee aging is directly related to behavioral state. The non-reproductive female worker caste exhibits a behavioral plasticity; termed “temporal polyethism,” where “nurses” transition to “foragers” in response to a multitude of environmental and physiological factors [13,14]. The pace of these transitions can be increased, decreased, or reversed by controlling these cues [15].

Typically, during the first 2–3 weeks of adult life, female workers perform hive maintenance and brood care, or nursing, during which they rarely fly. After transitioning to foraging, workers can fly long distances (up to 8 km) gathering nectar and pollen for several hours per day [16]. Once honey bees begin to forage for pollen and nectar, their aerobic metabolism greatly increases. Foraging honey bees have a metabolic rate of 100–120 mL O_2_ g^−1^ h^−1^, which is the highest mass-specific metabolic rate known and is 10–100 times higher than in nurse bees, which fly much less often [17]. Previous experiments from our laboratory suggest that the elevated metabolism of flying honey bees likely produces high levels of ROS that, coupled with an age-dependent decrease in antioxidant activity, negatively affects longevity [18]. Additionally, foraging bees show a decline in flight capacity with age [19] and time spent flying is negatively correlated with survivorship [20]. When the transition from nurse to forager is delayed, bees that stay in the hive can live up to 8 times longer than bees that transition naturally [21].

Another aspect of the plasticity of honey bee aging is the ability to revert from foraging tasks to in-hive nursing duties [22,23], with an accompanying reversion of many physiological characteristics. During behavioral reversion, hypopharyngeal glands (which produce food that young larvae consume and that atrophy in foraging bees) redevelop [24,25], juvenile hormone titers drop [22,23], and vitellogenin levels increase [26]. Reverted nurse bees also undergo a reversal of the immunosenescence observed in foraging bees [24]. Foragers’ age-related learning deficits also reverse during behavioral reversion [27]. Despite exhibiting many of the physiological traits of typical nurse bees, reverted nurse bees are not identical to typical nurse bees. Reverted nurse bees have a mixed proteomic profile that is similar to both nurses and foragers [28]. Additionally, some foragers appear to reach a threshold where they are unable to revert and continue to progress towards functional senescence [28]. It remains unclear if reverted nurse bees revert at the genomic level or if the effects of extended flight bouts on gene expression are permanent.

Here we examine how performance of behaviors with low *vs.* high metabolic cost affects gene expression in the flight muscle and the brain as bees transition to foraging and during reversion from forager to nurse. We chose to do this analysis on both tissues because brain tissue is particularly susceptible to oxidative stress [29] and flight muscle experiences oxidative stress resulting from flight [18]. In these experiments, we compared whole-genome transcriptional profiles of nurse bees and forager bees of different ages with different flight histories, including reverted nurse bees. We found that patterns of transcription differ between tissues in response to flight and that these changes can partially revert. We identified particular transcripts involved in stress response pathways that are differentially expressed between bees of various ages and with various fight experiences.

## 2. Experimental Section

### 2.1. Field Methods

Honey bees (*Apis mellifera* L.) used for this project were reared at the University of Nevada, Las Vegas apiary. For the microarray experiments and follow-up mRNA quantification of immune genes, 4 source colonies headed by unrelated single-drone inseminated queens (Glenn Apiaries, Fallbrook, CA, USA) carrying the Minnesota (MN) varroa sensitive hygienic (VSH) genotype were used to obtain age/genotype-matched workers to start single-cohort colonies (SCCs). Single, drone-inseminated queens were chosen to head the source colonies for the microarray experiment to decrease the genetic variability between worker bees. From these source colonies, eight SCCs were created using a frame of pollen, an open frame for the SCC’s queen to lay in and a frame of honey. Each SCC contained approximately 2,000 bees originating from multiple source colonies. A SCC uses skewed colony age demography to induce precocious foraging in approximately 10% of the bees at 7–10 days of age, whereas the onset of foraging normally takes place at 21–25 days of age [30]. The SCC allows the effects of age and behavior to be decoupled, permitting comparison of same-aged bees with drastically different flight histories and bees of different ages with the same behavioral activity.

Reversion colonies were made from 4 of the original SCC colonies (3 in June and 1 in July). Reversion colonies were made by collecting 1,000–3,000 19–22 day old foragers from a SCC colony’s entrance and placing that colony’s foragers into a new nucleus hive containing one frame of open larvae (1st–5th instar) with occasional capped brood, one frame each of honey and pollen, one sugar water in-hive feeder (half filled), a small pollen patty, and a Bee Boost strip at the center as source of queen mandibular pheromone. Foragers from separate colonies were not mixed together. This was done for each of the 4 colonies in turn resulting in 4 reversion colonies. The original colonies and their queens were moved out of the bee yard. The reversion colonies were moved to the location of the parent colonies and kept closed for three days to keep the foragers inside and force some to revert. Although unconventional, we chose to confine bees to the colony for three days to induce more bees to detectably revert (as suggested by Zachary Huang pers. comm.). Using our altered methodology, more foragers reverted and reverted nurses were more clearly attending to brood compared to our pilot study, which used previously described methods that do not include confinement [24,27,28]. This set of manipulations induced a small portion of the foragers in each of the 4 colonies to revert to nursing tasks [22,23,31]. Reverted nurse bees were then identified and collected. 

In a separate follow-up experiment, additional SCCs were made to measure mRNA levels in genes known to play a role in aging [32,33,34]. Two source colonies headed by naturally-mated queens were used to obtain workers to start four SCCs. Each SCC contained bees from multiple colonies. Naturally-mated queens were used in this experiment to mimic typical hive conditions, which naturally have high genetic variability. Because the SCCs for the experiment above were made from only four source colonies headed by SDI queens, those colonies would represent at most four patrilines and four matrilines. In contrast, these SCCs made from source colonies with naturally mated queens like had 10–40 times more genetic variability than SCCs in the first experiment. The SCCs were created using seven frames of brood from the source colonies. The frames were placed in an incubator (32 °C, 75% relative humidity RH, 24 h dark cycle) and newly-eclosed bees were removed from the frames every 24 h. A SCC was formed by housing approximately 2,000 single, day-old workers from, which eclosed over two consecutive days, with a naturally mated queen (Koehnen Apiaries, CA, USA). These bees were placed in a nucleus colony containing one frame each of pollen and honey and three empty frames to allow the queen to lay eggs. The dorsal thorax of each bee was marked with a single dot of paint (Testors, Rockford, IL, USA) to indicate age, prior to placing them in their SCC. Each SCC was kept in the laboratory for five days post-adult emergence to allow for young worker adult maturation and queen egg laying before being moved to the outdoor apiary on the UNLV campus.

### 2.2. Behavioral Groups

Foraging bees were identified as bees returning to the hive with a pollen load or distended abdomens from nectar. Nurse bees were identified as individuals placing their heads into frame cells containing an egg or larva [13]. Once identified behaviorally, bees were marked with a dot of paint, and only marked foragers and nurses were used for these analyses. Sample sizes collected were n = 5–6 per group for the microarray and immune gene experimental colonies and n = 12 per group for the aging gene experiments. Bees were immediately placed in liquid nitrogen upon collection and stored at −80 °C until processing so that mRNA levels accurately reflected natural differences in gene expression.

To separate the effects of age and behavior, nurse bees and forager bees were collected at various time points (8–10 days, 19–22 days, and 25–26 days) with various flight experiences ranging from less than one day of flight experience to over 25 days (See Table 1 for more details). For the aging genes experiment, the same collection regime was followed except collection days for nurses and foragers were extended until 40 days past eclosion so that any pronounced changes in gene expression (mRNA levels) could be detected. Because this second experiment was concerned with expression of genes involved in aging, we did not create reversions. During all experiments, capped brood was removed and replaced with empty frames to encourage the queen to lay eggs normally and to prevent any new bees from eclosing and changing the demography of the SCCs.

**Table 1:** Behavioral Groups. Group of nurse bees and forager bees with varying amounts of flight were used in the microarray and real-time PCR experiments. Abbreviations are used throughout experiments.

Behavioral Group	Abbreviation	Age (days)	Days of flight
Young nurse	YN	8 to 10	< 1
Precocious forager	PF	8 to 10	2 to 3
Older nurse	ON	19 to 22	< 1
Forager-low flight	TL	19 to 22	2 to 3
Forager-high flight	TH	19 to 22	7 to 9
Forager-old	OF	25 to 26	10 to 12
Reverted nurse	RN	25 to 26	7 to 9

### 2.3. RNA Extraction

Whole bee heads and thoraces were partially lyophilized at −70 °C to facilitate dissection [35]. Heads and thoraces were then dissected on dry ice to prevent RNA degradation. Care was taken to avoid including the hypopharyngeal or subesophageal glands in brain samples (leaving the optic lobes, antennal lobes, and mushroom bodies) and to precisely obtain the primary flight muscles of the thoraces. The high yields of RNA needed for microarray experiments were extracted from dissected brains and thoraces using PicoPure RNA Isolation kits according to the manufacturer’s protocol (Molecular Devices, Sunnyvale, CA, USA). For the aging genes real-time PCR experiment, which required less RNA than the microarray experiment, RNA was extracted using 1 mL of Trizol reagent (Invitrogen, Carlsbad, CA, USA) following the manufacturer’s protocol.

### 2.4. Microarray Hybridization

**Figure 1:**
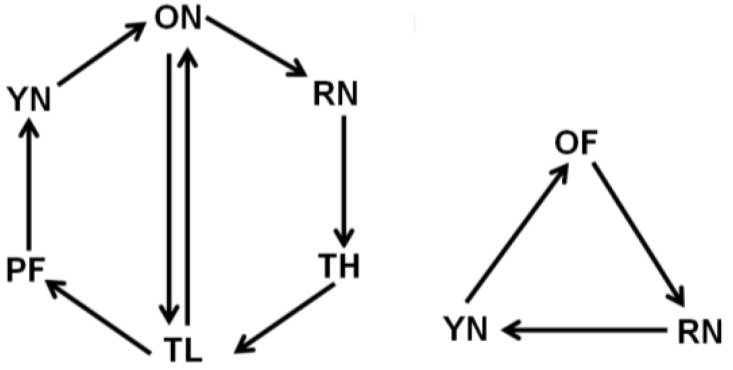
Microarray experimental design. The microarray experiment was designed with 132 arrays in a “round-robin” style. Each arrow represents 6 arrays. **YN** = young nurse (10 days-old; <1 day flight); **RN** = reverted nurse (25–26 days-old; 7–9 days flight); **OF** = old forager (25–26 days-old; 10–12 days flight); **TH** = typical-aged forager-high flight (19–22 days old; 7–9 days flight); **ON** = older nurse (19–22 days old; <1 day flight); **TL** = typical-aged forager-low flight (19–22 days old; 2–3 days flight); **PF** = precocious forager (8–10 days old; 2–3 days flight).

Brains and thoraces from the same individuals of all behavioral groups were compared on a total of 132 arrays. The samples were hybridized against each other using a loop design [36] (See Figure 1 for experiment design). The microarray hybridization procedure followed previously described methods [37]. Extracted total RNA, cDNA, and labeled aRNA sample concentrations were quantified using a Nanodrop ND-1000 spectrophotometer (NanoDrop Technologies, Wilmington, DE, USA). Five hundred nanograms of RNA were amplified using the MessageAmp II aRNA amplification kit (Ambion, Austin, TX, USA). For each individual, 4 micrograms of brain RNA were labeled with cy3 and 4 micrograms of thorax RNA were labeled with cy5 using a Kreatech Labeling Kit (Applied Biosystems, Salt Lake City, UT, USA). The directionality of the dye labeling was switched between replicates to avoid a dye bias. Whole genome oligonucleotide arrays (W.M Keck Center for Comparative and Functional Genomics, University of Illinois, Urbana-Champaign) were hybridized with 120 picomoles of each labeled tissue’s probes. Arrays were then scanned with a GenePix scanner (Molecular Devices, Sunnyvale, CA, USA) and visualized using GenePix software (Agilent Technologies, Santa Clara, CA, USA). Prior to data analysis, each array was visually inspected for any inconsistencies in dye incorporation.

### 2.5. Microarray Data Analysis

Analysis was implemented in R version 2.8 [38]. Data was normalized using the Limma package in R [39]. Spots were log-transformed and within-slide normalization was conducted using a print-tip Loess to correct for differences between print-tips on the array printer and variation during the print run [39]. No background correction was used for within-slide normalization. For between-slide normalization, a quantile normalization method was used [40]. Pre- and post- normalization MA plots were generated to ensure each array was of acceptable quality to use. To detect differential expression, we used a mixed-model two-way ANOVA from the MAANOVA package in R [41,42]. The model was fit with treatment and dye as fixed effects and array as a random effect. Contrast statements were used to identify transcripts that were differentially expressed between different tissues. Transcripts were considered differentially expressed when their interactions yielded an adjusted p-value (FDR) of less than 0.05 [43]. Gene ontology analysis was conducted using ArrayTrack [44]. The microarray data for this manuscript were submitted to the NCBI Gene Expression Omnibus database (accession number: GSE40650).

### 2.6. Quantitative Real-Time Polymerase Chain Reaction

We performed quantitative real-time PCR on flight muscle for 6 immunity genes and on brains and flight muscle for 3 aging genes that yielded statistically significant results in the microarray experiments. For the immunity genes, cDNA was synthesized from 200 ng of RNA used for the microarray experiments and for the aging genes, 200 ng of RNA was used following manufacturer’s instructions using a qScript cDNA synthesis kit (Quanta Biosciences, Gaithersburg, MD, USA). qRT-PCR was performed on an iCycler iQ (BioRad, Richmond, CA, USA) using the SYBR green detection method (Quanta Biosciences, Gaithersburg, MD, USA). Five individual thoraces were used for the immunity gene analysis and brains and thoraces from five different individual bees were used for the aging genes experiment. As a transcriptional control, mRNA levels of the genes of interest were measured relative to the housekeeping gene, ribosomal protein 49 (*rp49*). Statistical analyses were done using delta Ct values using a two-way analysis of variance with age and behavior as the main effects and colony and PCR plate as random effects in JMPv8.0 (SAS Institute, Cary, NC, USA).

## 3. Results and Discussion

### 3.1. Behavior Induces Dramatic Changes in Global Gene Expression of *A. mellifera* Brains and Thoraces

**Figure 2:**
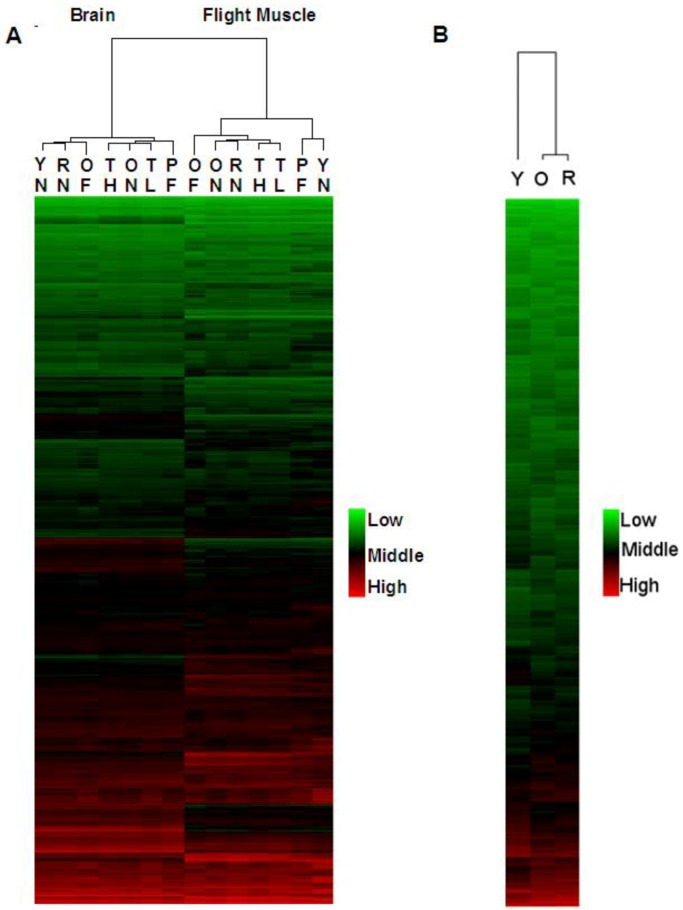
Global expression analysis. Hierarchical clustering was performed to visualize global patterns of gene expression. Replicates were averaged before analysis. Each graph represents over 12,000 transcripts and each row of the graphs represents one transcript. (**A**) Unique patterns of transcription were seen between groups that represented various ages and behavioral groups with varying amounts of flight experiences. Unique expression patterns were also seen between tissues. (**B**) Hierarchical clustering reveals groups more closely related in age are most related. **YN** = young nurse (8–10 days-old; <1 day flight); **RN** = reverted nurse (25–26 days-old; 7–9 days flight); **OF** = old forager (25–26 days-old; 10–12 days flight); **TH** = typical-aged forager-high flight (19–22 days old; 7–9 days flight); **ON** = older nurse (19–22 days old; <1 day flight); **TL** = typical-aged forager-low flight (19–22 days old; 2–3 days flight); **PF** = precocious forager (8–10 days old; 2–3 days flight). **Y** = young bees (8–10 days-old); **O** = old bees (25–26 days-old foragers; 19–22 day-old nurses); **R** = reverted nurse bees (25–26 days-old; 7–9 days flight).

In this study, we compared age-matched nurse bees and forager bees with differing amounts of prior flight activity to examine gene expression associated with flight in brain tissue and flight muscle. Using whole-genome oligonucleotide arrays, either 6 or 12 biological replicates (Figure 1) were used for each group involved in this analysis. By using a manipulation that causes forager bees to revert back to nurse bees, we were able to determine the reversibility of the transcriptional profile of these bees (Figure 2A). Groups more closely related in age had the most similar transcriptional patterns (Figure 2B). Similar to the findings of a previous study looking at brain transcriptional patterns [45], we found that honey bee flight induces unique patterns of expression across the genome in brain tissue, but in the flight muscle we saw age-related patterns of expression similar to expression patterns in *Drosophila* thoraces [46].

In brain tissue (Figure 2A), gene expression patterns between young, aged-matched (8–10 days old) nurses and foragers were the least similar. These expression differences were not as pronounced between older nurses (19–22 days old; <1 day flight experience) and foragers (19–22 days old; 2–3 days flight experience or 7 to 9 days flight experience). In flight muscle (Figure 2A), reverted nurse transcriptional patterns were most related to older nurses (19–22 days old; <1 day flight experience) followed by typical-aged foragers (19–22 days old with either 2–3 days flight experience or 7 to 9 days flight experience). We found no effect of the number of days spent flying, independent of age, in either tissue. This suggests that the transition to foraging behavior and the interaction between age and frequent flight have more impact on gene expression and senescence than the actual amount of flight. Over the course of an entire foraging day, acute effects of flight have an effect on levels of heat shock proteins and antioxidant activity, but behavioral state (nurse or forager) does not have an effect [18]. Consequently, behavioral state affects many transcripts, such as those involved in signaling pathways. Because no transcriptional changes between high and low flight foragers were detected, but changes between age-matched nurses and foragers were present, this suggests the transcriptional differences are due to both flight and caste-specific behavioral/physiological differences. To fully separate acute effects of flight and effects of physiology/behavior, nurse bees with high amounts of flight time would be ideal. However, obtaining these bees in a natural hive or SCC is difficult and likely unfeasible at a large scale. To more closely examine the effects of flight, our ongoing studies are examining foragers restricted from flying, foragers with high and low amounts of flight time, and nurse bees. Longevity in honey bees is directly related to flight activity [47]. As the transition from in-hive nursing tasks to outside foraging tasks occurs, senescence accelerates, and time spent foraging is negatively correlated with survivorship [20]. After approximately 14 days of foraging, foragers experience cognitive decline [48] and oxidatively-damaged proteins accumulate in the brain [49]. As our lab has previously shown, a decrease in antioxidant activity also occurs in forager and nurse flight muscle, but not the brain, of bees greater than 30 days of age [18]. As foraging time increases bees show a decline in flight capacity [19]. However, longevity increases when the transition from nurse to forager is delayed [21]. Together, the results of previous studies and the results from this study suggest that brain tissue and flight muscle respond differently to cellular stress. These results also imply that the brain is perhaps more effectively able to mitigate flight associated cellular stress. Flight muscle is the most metabolically active tissue in the honey bee [17]; hence it experiences greater levels of ROS production. As a result, the honey bee’s ability to mitigate stress in this tissue may become compromised as foraging time increases. Indeed our previous work suggest older foragers are less able to mitigate that stress [18]. Once the flight muscle is compromised, the honey bee forager is essentially ecologically dead even if the brain is not yet showing signs of senescence. Because brain tissue is particularly susceptible to stress [29], the honey bee brain may possess additional mechanisms to mitigate flight-associated stress that are absent in flight muscle. However, even protective mechanisms in the brain may be eventually overcome leading to cognitive or whole-organism functional senescence.

Previous studies show that under certain conditions forager bees can behaviorally revert and return to nurse behaviors [22,23] and that this reversion is also accompanied by physiological reversions. During reversion, JH levels drop [22,23], vitellogenin levels increase [26], hypopharyngeal glands redevelop and immunosenescence reverses [24]. In general, our results reveal that, at the transcriptional level, there also exists a reversion, but some aspects of forager gene expression patterns persist (Figure 2A). This intermediate pattern of transcription is likely due to the combined effects of both behavioral/physiological differences between nurses and foragers as well as the effects of flight. In brain tissue, reverted nurse transcriptional patterns are most closely related to those of young nurses (8–10 days old; <1 day flight experience). However, in flight muscle, reverted nurse transcriptional patterns were most related to older nurses (19–22 days old; <1 day flight experience) followed by typical aged foragers (19–22 days old with either 2–3 days flight experience or 7 to 9 days flight experience). This result suggests the brain has more transcriptional plasticity than flight muscle, which may allow the brain to more effectively mitigate stress.

After a small pilot study using the reversion methods of previous studies [22,23,24,27,28] yielded only a small number of reverted foragers exhibiting clear nursing behavior , we chose to modify the reversion method as noted above. Our modified method used queen pheromone in place of a queen, and confinement of foragers to the hive during the reversion time. This strategy induced more frequent and less ambiguous reversion perhaps because closing the colony blocks out photoperiod, allowing the reverted foragers to more easily assume a more nurse-like circadian rhythm. Despite these differences, our transcriptional data agree with Wolschin *et al*. [28], which show the proteomic signature of reverted nurses is a mix between nurses and foragers. Additionally our transcriptional data and the proteomic data of Baker *et al*. [27] show reversion affects levels of cellular stress transcripts or proteins. Because of experimental agreement between our results and others, we conclude that our reversion technique and collection regime, albeit slightly different than other reversion techniques [23,24], produced a comparable reversion. While it is possible that confining foragers to the hive during reversion induced a stress response that altered transcriptional patterns, this response may also be present in other reversion techniques as reversion may be a response to colony level stress. Additionally, Baker *et al*. [27] and Wolschin *et al*. [28] allowed the reversion to continue longer than three days before collection. Thus, it is possible that some effects of reversion that do not occur immediately were undetectable in this study.

### 3.2. Unique and Shared Transcripts between Behaviors and Ages

**Figure 3:**
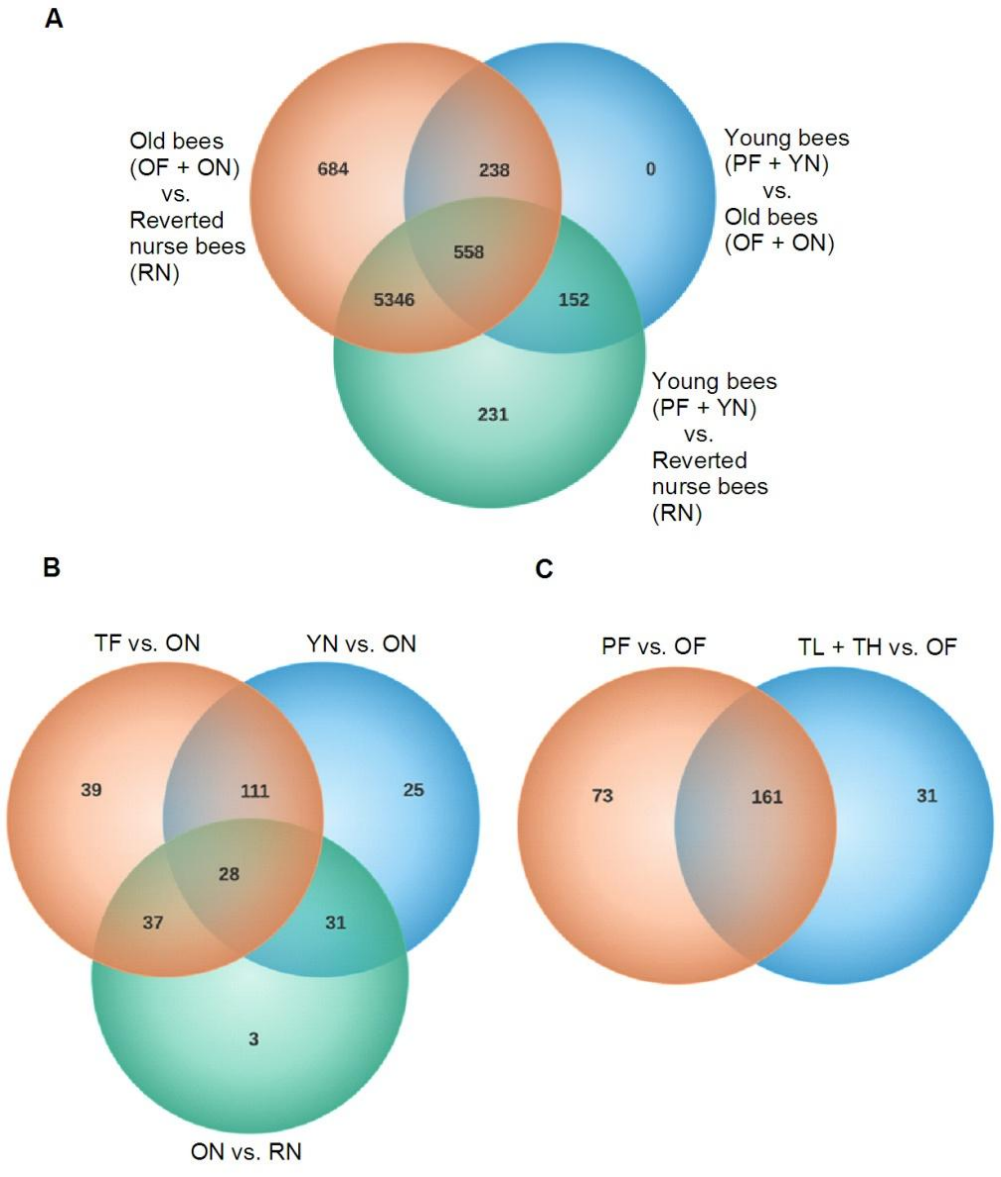
Unique and shared transcripts. Venn diagrams were used to visualize transcripts unique and shared between each group. Numbers represent transcripts differentially expressed (FDR < 0.05). (**A**) Transcripts differentially expressed between young bees, old bees and reverted nurse bees. (**B**) Transcripts differentially expressed between nurse bees. (**C**) Transcripts differentially expressed between forager bees. **YN** = young nurse (8–10 days-old; <1 day flight); **RN** = reverted nurse (25–26 days-old; 7–9 days flight); **OF** = old forager (25–26 days-old; 10–12 days flight); **TH** = typical-aged forager-high flight (19–22 days old; 7–9 days flight); **ON** = older nurse (19–22 days old; <1 day flight); **TL** = typical-aged forager-low flight (19–22 days old; 2–3 days flight); **PF** = precocious forager (8–10 days old; 2–3 days flight).

We used a two-way ANOVA to find transcriptional differences between young bees, older bees and reverted nurse bees as well as between different aged nurses and between different aged foragers. Statistically significant differences in transcript expression were found by using the false discovery rate for multiple comparisons. Figure 3A shows which differentially expressed transcripts were unique to each age comparison and which transcripts overlapped. We found 684 differentially expressed transcripts unique to young bees (PF and YN) compared to old bees (OF and ON) and 231 transcripts unique to young bees (PF and YN) compared to reverted nurse bees (RN). This result implies only a small subset of the genes differentially transcribed during the nurse-to-forager transition do not revert during reversion from forager to nurse. There were no unique transcripts when old (OF and ON) bees were compared to reverted nurse bees (RN). When we analyzed what transcripts were unique within nurses and within foragers (Figure 3B,C), we found that foragers had more differentially expressed transcripts than aged-matched nurses, indicating that gene expression varies with age and behavior but flight has a greater effect on gene expression than age. This result is not surprising because flight produces high levels of reactive oxygen species and flight muscle antioxidant capacity decreases with age [18], both of which may lead to changes in gene expression.

### 3.3. Gene Ontology Analysis Reveals Age Causes a Decrease in Certain Stress and Immune Processes in A. mellifera

**Figure 4:**
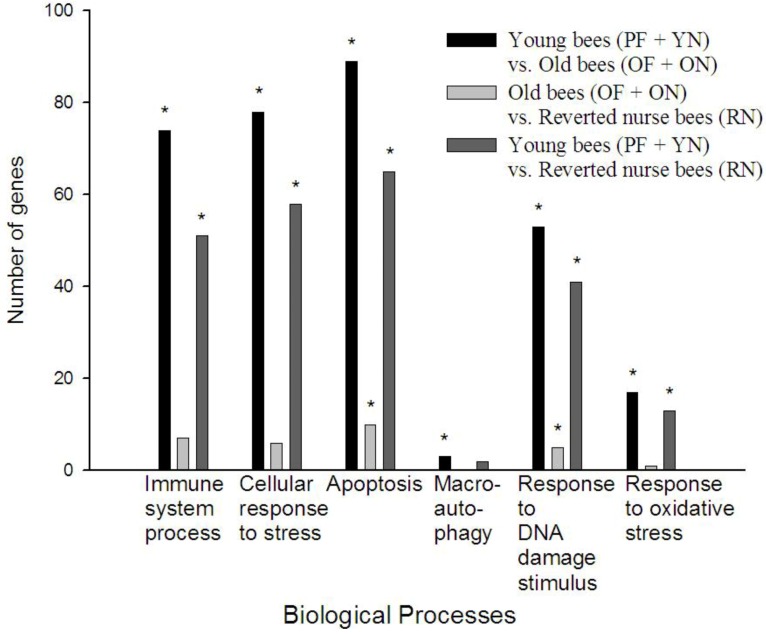
Comparison of biological processes. Gene ontology analysis revealed a series of biological processes involved in immune function and aging/senescence that were enriched in each comparison. The x-axis represents the number of genes differentially expressed for the biological processes represented on the y-axis. Stars represent a Fisher p-value <0.05. **YN** = young nurse (8–10 days-old; <1 day flight); **RN** = reverted nurse (25–26 days-old; 7–9 days flight); **OF** = old forager (25–26 days-old; 10–12 days flight); **ON** = older nurse (19–22 days old; <1 day flight); **PF** = precocious forager (8–10 days old; 2–3 days flight).

To put the changes in gene expression we identified into a functional context, we performed a gene ontology and biological pathway analysis in ArrayTrack. Figure 4 shows six biological processes relevant to immune function and aging/senescence. In general, young bees compared to old bees had the most differentially expressed transcripts involved in any particular biological process, while young bees (PF and YN) compared to reverted nurse bees (RN) had considerably less differentially expressed transcripts between each other, if any at all. All three comparison groups yielded statistically significant enrichment of response to DNA damage stimulus (GO:0006974) and apoptosis (GO:0006915).

In our specific comparison of old bees (ON and OF) *vs.* reverted bees (RN), we found two gene ontology categories, apoptosis (GO:0006915) and response to DNA damage stimulus (GO:0006974), statistically enriched. These results may suggest that reversion from forager to nurse may increase DNA damage repair thus decreasing apoptosis and increasing lifespan. In *Drosophila*, apoptosis increases with age, limiting lifespan [50], and so honey bees may employ a mechanism during behavioral reversion that could decrease apoptosis to delay functional senescence. Additionally, macroautophagy (GO:0016241) was significantly enriched in young (PF and YN *vs.* old bees (OF and ON) but not in any other comparisons. Under most cellular conditions, autophagy promotes cell survival by adapting the cell to various conditions of stress. However, if autophagic activity is lost, cell death is accelerated leading to functional senescence [51]. The transition to foraging behavior contributes to a loss of autophagy, thus increasing senescence in the honey bee.

### 3.4. KEGG Pathway Analysis Reveals Age Alters Specific Signaling Pathways in A. mellifera

**Figure 5:**
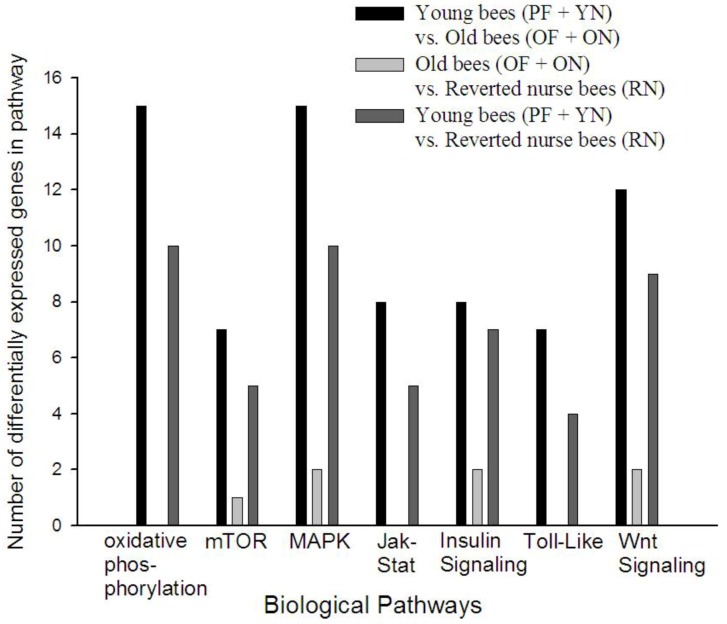
Comparison of signaling pathways. The KEGG database revealed pathways and the number of differentially expressed (FDR < 0.05) transcripts. The x-axis represents the number of genes differentially expressed for the biological pathway represented on the y-axis.**YN** = young nurse (8–10 days-old; <1 day flight); **RN** = reverted nurse (25–26 days-old; 7–9 days flight); **OF** = old forager (25–26 days-old; 10–12 days flight); **ON** = older nurse (19–22 days old; <1 day flight); **PF** = precocious forager (8–10 days old; 2–3 days flight).

Along with the gene ontology analysis, we conducted a biological/biochemical pathway analysis to putatively identify pathways that differed in the expression of their constituents between age/behavioral groups (Figure 5). The analysis was implemented in ArrayTrack using the KEGG database of pathways. Similar to biological processes, young bees compared to old bees had the most differentially expressed transcripts in each pathway, while young bees compared to reverted nurse bees had far fewer differentially expressed transcripts and in some cases none at all. The oxidative phosphorylation, Jak-Stat, and Toll-like receptor pathway signaling pathways were not represented in old bees (OF and ON) compared to reverted nurse bees (RN), but pathways such as MAPK and mTOR were represented. These results suggest a number of distinct biochemical pathways are activated or repressed with age and the reversion from forager to nurse.

### 3.5. Expression of Transcripts Involved in the A. mellifera Immune Response

As nurse bees transition to foraging, systemic levels of JH increase [52], and this increase in JH is correlated with a dramatic loss of hemocytes [26,53,54]. With the loss of functional hemocytes, the nodulation response, a key aspect of the insect infection response, is lost [55,56]. Levels of both JH and hemocytes are restored during reversion of foraging bees to nurse bees [24]. These results suggest that loss of immune function may contribute to functional senescence in foraging honey bees. In a study measuring expression of a variety of immunity transcripts in adult worker bee abdomens Evans *et al*. [57] found 6 immunity transcripts were highly up-regulated following injection of pathogens or wounding, and 25 immunity transcripts remained unchanged. A bee’s status as a forager or nurse, therefore, could be more important in determining immune function than its age because as intensity of flight activity increases, proper immune responses may decrease. 

**Figure 6:**
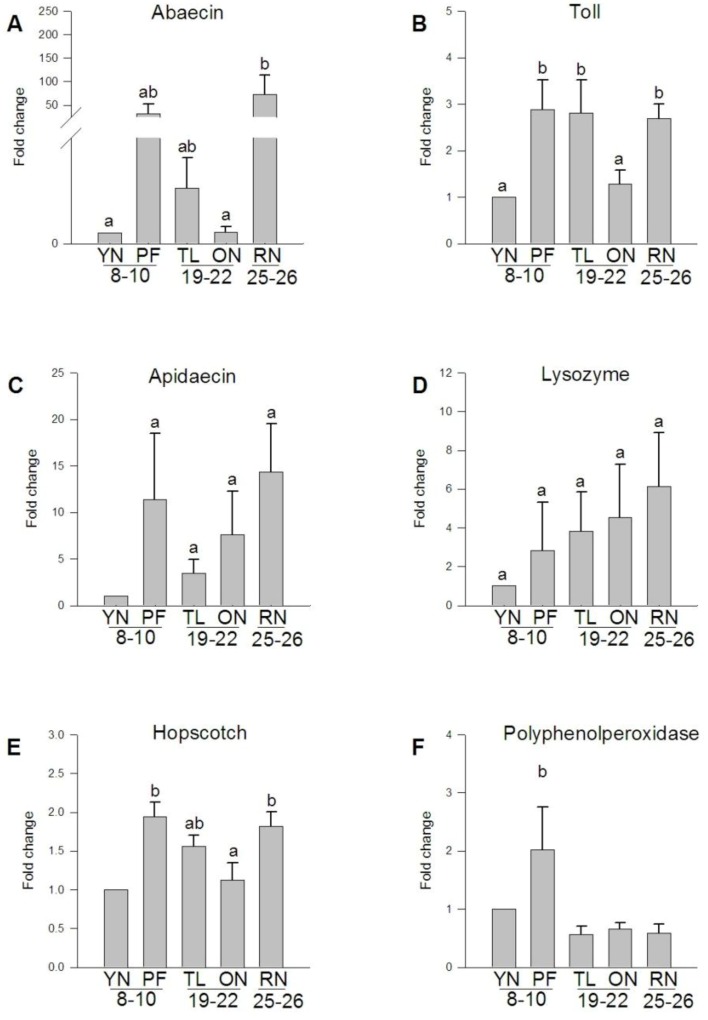
Expression of immune transcripts in *A. mellifera* flight muscle. Quantification ofmRNA levels of bee specific immune transcripts (**A**, **C**), transcripts involved in immune signaling pathways (**B**, **E**), and innate immune response transcripts (**D**, **F**) revealed differential expression between ages and behaviors. Fold change represents the relative difference compared to one day old bees.

We determined whether different amounts of flight and behavioral reversion have an effect on the expression of a subset of these immunity transcripts identified as differentially expressed in our flight muscle microarray data (Figure 6). We measured mRNA levels in 2 bee-specific immunity transcripts: apidaecin (*apid1*) and abaecin (LOC406144), 2 immune signaling pathway transcripts: Toll-like receptor (*tlr1*) and hopscotch (*hop*), which is part of the Jak-Stat signaling pathway, and 2 transcripts involved in insect innate immunity: lysozyme (*lys1*) and polyphenoloxidase (*ppo*) using qRT-PCR. If the loss of immune function were primarily due to age, one would expect to see decreased expression of all of these immune function genes with aging in nurses as well as foragers.

Expression of apidaecin mRNA (Figure 6A) was similar between bees performing different behaviors regardless of age and no statistically different expression patterns by age or behavior alone were apparent (Figure 6C). Expression of both transcripts involved in immune signaling pathways (toll-like receptor and hopscotch) was up-regulated with the transition to foraging and reverted nurse bee expression levels were more similar to those of forager bees than to nurse bees (Figure 6B,E). Expression of lysozyme did not statistically differ with age or behavior (Figure 6D). As foraging flights increased, mRNA levels of polyphenoloxidase decreased and remained lower when reverted bees returned to in-hive nursing tasks (Figure 6F).

These data suggest that for nurse bees, age is not the primary influence on the expression of these transcripts in flight muscle. Although the expression of most immune transcripts did not change with age, some transcripts in flight muscle were behaviorally up-regulated as nurses transitioned to foragers. This transition increases levels of ROS in the flight muscle as both metabolic rate and metabolic capacity increase [58]. In mammals, reactive oxygen species stimulate innate immunity signal transduction pathways [59], such as the toll-like receptor signaling pathway [60]. Because honey bees have activity rates and levels of endothermy more similar to mammals than most insects, [61,62] this immune pathway upregulation may also occur in bees and other active, strongly endothermic insects. In all of the immune transcripts we measured, reverted nurse bees had mRNA levels similar to that of foragers, suggesting transcripts involved in the immune response may not revert during transition from forager to nurse. Another explanation for absence of detectable changes in reverted nurse bees may that reverted nurse bees were collected 3 days post-reversion and the transcriptional control mechanisms of immune transcripts in honey bee flight muscle may require greater than 3 days to revert. In *Drosophila*, expression of immunity genes increase with age [63], but honey bee immune gene transcription appears to be more tied to typical behavioral trajectories rather than age. Although reverted nurses experience a physiological reversion (enough to care for and feed young larvae), this was accompanied only by a partial genomic reversion. The linked pressures of intense flight metabolism and ROS production in foragers may lead to a loss of immune function that is delayed, but not prevented, by the reversion process. Therefore, the longevity conferred upon older nurses restricted to the hive [21] may be more generous than that accorded to reverted nurses that once foraged.

### 3.6. Expression of Longevity Mediating Transcripts

**Figure 7:**
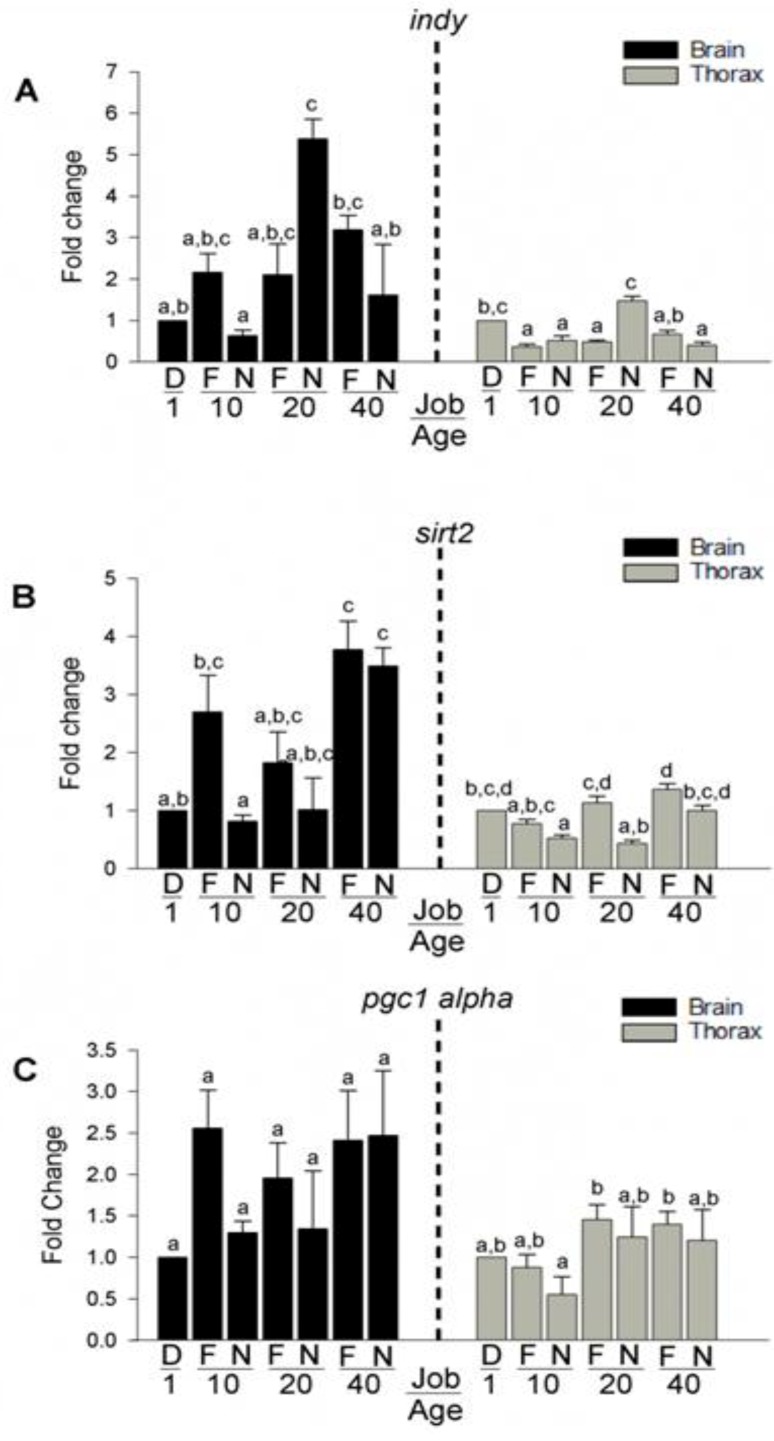
Expression of longevity mediating transcripts in *A. mellifera* flight muscle and brain.mRNA levels of putative genes involved in aging/senescence were measured in brain tissue and flight muscle. Expression of (**A**) *indy*, (**B**) *sirt2*, and (**C**) *pgc1alpha* revealed distinct expression patterns both between tissues and within tissues. D = single day old bee; N = nurse; F = forager. Fold change represents the relative difference compared to one day old bees.

A large body of the research on honey bee longevity and senescence has focused on a few main factors such as vitellogenin expression and insulin signaling [64]. These data from our microarray experiment suggests additional factors for investigation. To further explore the role of aging-related genes identified in the GO analysis relative to behavior and age, we set up another SCC experiment and obtained age-matched nurse bees and forager bees at days 10, 20 and 40 post eclosion. We quantified brain tissue and flight muscle mRNA levels of 2 transcripts differentially expressed in our array analysis and known to mediate longevity in other model organisms such as flies and nematodes, I’m not dead yet (*indy*), silent mating-type information regulation 2 (*sirt2*) (Figure 7). We chose an additional transcript not on the array but identified in other model organisms involved in regulating mitochondrial biogenesis, Peroxisome proliferator-activated receptor gamma coactivator 1-alpha (*pgc1alpha*) which may be intimately connected with honey bee metabolism and senescence (Figure 7).

Although controversial, decreased activity in the *indy* gene, a transporter of Kreb’s cycle intermediates, extends lifespan in *Drosophila* [34]. Expression of *indy* (Figure 7A), in both the brain and flight muscle, significantly increased in nurse bee mRNA between 10 days of age and 20 days, and then decreased to levels seen at 10 days by 40 days of age. However, single day old bees had mRNA levels similar to 20 day old nurse bees. These data are consistent with studies that show delaying the onset of foraging and remaining a nurse bee extends lifespan [21]. We did not see an effect of the transition to foraging on expression of *indy* in either brain tissue or flight muscle. This result suggests that the transition to foraging does not affect levels of *indy*, negating the potential protective effects of its decreased activity.

In *Drosophila* increased levels of the NAD dependent histone deacetylase, *sirt2*, increased lifespan [32]. In our study, expression of *sirt2* (Figure 7B) levels in the brain revealed precocious foragers had higher levels of *sirt2* compared to their age-matched nurse-bee counterpart. There was also an age-related increase in nurse bees. In flight muscle, we saw an age-related increase in *sirt2* mRNA in both foragers and hive bees. Earlier up-regulation of *sirt2* in brain tissue may contribute to the brain’s ability to mitigate flight-associated cellular stress better than flight muscle, which up-regulates *sirt2* later.

Because honey bee flight is metabolically intensive [17], high levels of ATP are needed during this behavior. However, mitochondrial activity has been shown to decrease with age in *Drosophila* [65]. In contrast, honey bee flight occurs towards the later part of the honey bee life cycle, thus a mechanism likely exists to maintain levels of ATP and mitochondrial activity. Hence, we measured levels of *pgc1alpha*, a gene involved in regulating mitochondrial biogenesis [33]. Expression of *pgc1alpha* (Figure 7C) in the brain did not significantly differ between age or behavioral groups. In flight muscle, however, young nurse bees (10 days old) had lower *pgc1alpha* mRNA levels than that of 20 or 40 day old nurses. Expression of *pgc1alpha* in forager flight muscle did not significantly differ between age or behavioral groups. These results may imply functional senescence is delayed by maintaining steady levels of *pgc1alpha* expression in honey bee brain tissue and flight muscle. This is consistent with the findings of Williams *et al.* (in prep), which shows foragers and nurses, regardless of age, have similar activity levels of citrate synthase, an extremely robust enzyme that is routinely used to estimate mitochondrial number [66,67]. The impaired metabolic and flight capacity of aged honey bees may instead be due to structural lesions in the mitochondria and the sliding filament apparatus of flight muscle [68,69]; a possibility we are currently investigating.

## 4. Conclusions

Brain tissue and flight muscle responded differently to the cellular stress induced by flight. Our data suggest that intense flight (forager) *vs.* little-to-no flight (nurse) has a major effect on gene expression, rather than smaller-scale variation flight activity. The reversion from forager to nurse was also accompanied by a partial genomic reversion revealed by our microarray data, but the cellular and genomic mechanisms of how this reversion occurs remain unclear. Upregulation of *sirt2* in the brain during the transition from nurse to forager suggests a possible epigenetic mechanism of regulation and role for energy balance processes in honey bee senescence. Therefore, the behavioral and ecological contexts of bee flight should also be taken into account when considering the progression of honey bee senescence. Although honey bee flight likely produces high levels of ROS [16], which may be a contributing factor to senescence, this has not been demonstrated experimentally and the ultimate cellular cost of foraging has yet to be determined. Understanding how variation in the timing and duration of foraging leads to whole-organism functional senescence and what mechanisms are employed to prevent senescence in old and reverted nurse bees will be important in understanding honey bee aging and control of aging in general.

## Supplementary Files

Supplementary File 1
