# Supplementary material for: Effects of Flight on Gene Expression and Aging in the Honey Bee Brain and Flight Muscle
Source: Insects. 2012 Dec 20;4(1):9–30. doi: 10.3390/insects4010009 (PMC4553427; doi:10.3390/insects4010009)
Supplement: Supplementary File 1 [file insects-04-00009-s001.pdf]

All known genes only expressed in forager young vs. forager oldest.

Gene name	Description
LOC552406	similar to thyroid hormone receptor interactor 4
LOC550922	similar to mab-2 CG4746-PA
LOC724511	similar to zinc finger protein 239
LOC725732	similar to CG30492-PB, isoform B
LOC409141	similar to CG7272-PA
LOC410775	similar to wing blister CG15288-PB, isoform B
trxr-1	thioredoxin reductase
csnk1a1	similar to casein kinase 1, alpha 1
LOC726679	similar to CG5913-PA
LOC727045	similar to yellow-e2 CG17044-PA
LOC724136	similar to CG6305-PA
DWVgp1	DWVgp1
jhamt	similar to juvenile hormone acid methyltransferase CG17330-PA
LOC411021	similar to Ugt86Dc CG4739-PA
LOC725700	similar to vacuolar protein sorting 29 isoform 2
LOC412767	similar to CG2016-PB
LOC725149	similar to GPI7 protein
LOC551537	similar to enoyl Coenzyme A hydratase domain containing 3
Hydr2	similar to CG3488-PA
LOC411927	similar to CG15111-PB, isoform B
rpl21	similar to Ribosomal protein L21 CG12775-PA
LOC410487	similar to CG2446-PC, isoform C
LOC409573	similar to CG1832-PA, isoform A
Cpap3-c	similar to Gasp CG10287-PA
LOC411269	similar to CG14621-PA
LOC724457	hypothetical protein LOC724457
LOC409336	similar to CG4330-PA
GMCOX4	similar to CG9518-PA
LOC726400	similar to Fork head domain protein FD2
LOC410600	similar to Neu3 CG7649-PB, isoform B
LOC413274	similar to Guanylyl cyclase at 89Db CG14886-PA
rho-5	similar to rhomboid-5 CG33304-PA
CAP-D2	similar to Condensin complex subunit 1 (Chromosome condensation-related SMC-associated protein 1) (Chromosome-associated protein D2) (hCAP-D2) (XCAP-D2 homolog)
Dap160	similar to intersectin 2 isoform 3
LOC725547	similar to CG6131-PA

LOC551732	similar to Protein farnesyltransferase/geranylgeranyltransferase type I alpha subunit (CAAX farnesyltransferase alpha subunit) (Ras proteins prenyltransferase alpha) (FTase-alpha) (Type I protein geranyl-geranyltransferase alpha subunit) (GGTase-I-...
Mxp	similar to proboscipedia CG31481-PD, isoform D
LOC724611	similar to CG11323-PA
ras	similar to Inosine-5-monophosphate dehydrogenase (IMP dehydrogenase) (IMPDH) (IMPD) (Protein raspberry)
LOC725421	similar to CG7759-PB, isoform B
rtet	similar to tetracycline resistance CG5760-PA
LOC411412	similar to CG11665-PA
APP-BP1	similar to amyloid beta precursor protein binding protein 1
NT-12	similar to CG8291-PA, isoform A
LOC412815	similar to CG3523-PA
Mvl	malvolio
LOC724627	hypothetical protein LOC724627
LOC552061	similar to lemur tyrosine kinase 3
MRPL20	similar to mitochondrial ribosomal protein L20 CG11258-PA
KEL	similar to Ring canal kelch protein
LOC411564	similar to CG32645-PB
LOC552717	similar to CG5590-PA
LOC727381	similar to tRNA phosphotransferase 1 isoform 1
LOC552029	hypothetical LOC552029
LOC727201	similar to Carbamoyl-phosphate synthase [ammonia], mitochondrial precursor (Carbamoyl-phosphate synthetase I) (CPSase I)
LOC409207	similar to Synaptic vesicle membrane protein VAT-1 homolog

All known genes expressed only in forager old vs. forager oldest.

Gene name	Description
Sirt2	similar to Sirt2 CG5085-PA
jim	similar to jim CG11352-PC, isoform C
LOC409063	similar to black CG7811-PA
LOC408396	similar to rhea CG6831-PA
LOC725475	similar to COMM domain containing 8
LOC551270	similar to CG5222-PA
Mrjp1	major royal jelly protein 1
LOC411777	similar to CG17255-PA, isoform A
mGlutR2	similar to Metabotropic glutamate receptor 7 precursor (mGluR7)
Mrjp2	major royal jelly protein 2
LOC411476	similar to CG11505-PB, isoform B
LOC725607	similar to CG18292-PA
Mrjp5	major royal jelly protein 5
bib	similar to big brain CG4722-PA
Tpx-4	similar to Peroxiredoxin 2540 CG11765-PA
Med15	similar to Mediator complex subunit 15 CG4184-PA
LOC412235	similar to Caspase precursor (drICE)
LOC552787	similar to DNA topoisomerase 3-beta-1 (DNA topoisomerase III beta-1)
LOC726826	similar to Moca-cyp CG1866-PA, isoform A
LOC552065	similar to CG4785-PA

All known genes expressed only in nurse old vs. nurse oldest.

Gene name	Description
1790120	hypothetical LOC551946
1794082	similar to CG32645-PB
1790046	similar to Fork head domain protein FD2

All known genes expressed only in nurse young vs. nurse oldest.

Gene name	Description
LOC724457	hypothetical protein LOC724457
Mvl	malvolio
LOC725441	similar to DEAD (Asp-Glu-Ala-Asp) box polypeptide 49
LOC406097	integrin betaPS
LOC550655	similar to CG32066-PB, isoform B
LOC410775	similar to wing blister CG15288-PB, isoform B
LOC410272	similar to CG5535-PA, isoform A
nAChRa5	neuronal nicotinic acetylcholine Apisa7-2 subunit
CPR5	similar to CG1259-PB
LOC725547	similar to CG6131-PA
LOC724958	similar to Putative odorant receptor 13a
LOC724320	similar to transcription factor INI
LOC411908	similar to HLA-B associated transcript 5
LOC726127	similar to JTBR CG1935-PA

All known genes expressed only in nurse young vs. nurse old.

Gene name	Description
LOC725783	similar to CG11160-PA, isoform A
LOC409336	similar to CG4330-PA
LOC410600	similar to Neu3 CG7649-PB, isoform B
LOC412598	similar to CG18507-PA, isoform A
LOC725732	similar to CG30492-PB, isoform B
LOC411777	similar to CG17255-PA, isoform A
LOC552662	similar to CG11265-PA, isoform A
Tango10	similar to CG1841-PA, isoform A
Dap160	similar to intersectin 2 isoform 3
stnB	similar to Protein stoned-B (StonedB) (Stn-B)
LOC552333	similar to Organic anion transporting polypeptide 26F CG31634-PA
LOC726118	similar to CG30415-PA, isoform A
LOC412476	similar to CG7102-PA
LOC727045	similar to yellow-e2 CG17044-PA
rtet	similar to tetracycline resistance CG5760-PA
LOC411163	similar to Nat1 CG12202-PA
NT-12	similar to CG8291-PA, isoform A
LOC412065	similar to CG13646-PA
LOC411021	similar to Ugt86Dc CG4739-PA
LOC409969	similar to dihydrouridine synthase 3-like
5-HT1	similar to SERotonin/octopamine receptor family member (ser-4)
LOC551537	similar to enoyl Coenzyme A hydratase domain containing 3
Y-g	yellow-g
LOC410554	similar to methylcrotonoyl-Coenzyme A carboxylase 2 (beta)
LOC409395	similar to chromodomain Y-like protein 2
Lys-2	similar to CG11159-PA
LOC725759	similar to Peritrophin A CG17058-PA, isoform A
LOC726801	similar to CG8032-PA
LOC409573	similar to CG1832-PA, isoform A
LOC724776	hypothetical protein LOC724776
